# Ozone and Semen Quality: Berhane and Sokol Respond

**Published:** 2007-04

**Authors:** Kiros Berhane, Rebecca Sokol

**Affiliations:** Keck School of Medicine, University of Southern California, Los Angeles, California, E-mail: rsokol@usc.edu

We thank Bonde for his interest in our article (Sokol et al. 2006) and for drawing to our attention the literature on the effects of the welding occupation on male fertility. Although we agree with Bonde that the findings in the occupational studies he cited, for the most part, do not show a correlation between welding and abnormal semen parameters, one of his studies does report such an association (Bonde 1990), as does an article by Mortenson (1988). We find these data intriguing and puzzling, but we also would like to make the following points.

First, our study (Sokol et al. 2006) was population based and hence not directly comparable to the occupational studies.

Although our study directly investigated the effects of ozone, albeit from the ambient point of view and not via personal monitoring of exposure, the evidence from the occupational studies (Bonde 1990; Mortenson 1988) is an indirect and implied one. In these studies, direct O_3_ exposure information is not provided. In one of the negative studies (Hjollund et al. 1998), no differences in urine concentrations for the trace metals associated with welding were detected between welders and nonwelders, suggesting that “the negative results could be due to generally low exposure of the study base” (Hjollund et al. 1998).

The longitudinal design of our study (Sokol et al. 2006) gave us the opportunity to examine within-subject (over time) effects of O_3_ on male fertility in a sample that guarantees validity of the asymptotic inferences we made from the data.

The modeling techniques we used in the analysis have become fairly standard in analysis of longitudinal data such as ours; these techniques properly account for the within-subject correlation in the repeated measures for each subject. It is very unlikely that the O_3_ findings are artifacts of our modeling approach.

Finally, we carefully examined the potential confounding effects of weather, seasonality, and long-term time trends, and the O_3_ findings were robust to their inclusion in the models. Moreover, the O_3_ effects were robust to inclusion of other pollutants in the model.

That said, we readily acknowledge the excellent point that Bonde raised with respect to indoor–outdoor ratio of O_3_ exposure and possible misclassification of exposure due to the ambient nature of our exposure assignment. Ideally, we would have liked to assign direct personal exposure values or use a microenvironmental model (Navidi and Lurman 1995) to assign personal exposure values according to time–activity patterns, but this was not possible because of the retrospective nature of our study. However, we believe that the longitudinal design of our study (Sokol et al. 2006) gives us more confidence in the results, assuming consistent within-subject time–activity patterns.

We hope that future research will replicate our study (Sokol et al. 2006) in other locations around the world, preferably allowing for personal monitoring of exposure. We also hope that occupational studies will focus on direct assessment of O_3_ exposure to allow for direct comparisons with population-based studies whenever possible. Finally, we acknowledge that our epidemiologic findings of strong associations only add to the evidence in support of O_3_ effects on male fertility and but do not necessarily show causation.

## References

[b1-ehp0115-a0185b] Bonde JP (1990). Semen quality and sex hormones among mild steel and stainless steel welders: a cross sectional study. Br J Ind Med.

[b2-ehp0115-a0185b] Hjollund NH, Bonde JP, Jensen TK, Ernst E, Henriksen TB, Kolstad HA (1998). Semen quality and sex hormones with reference to metal welding. Reprod Toxicol.

[b3-ehp0115-a0185b] Mortensen JT (1988). Risk for reduced sperm quality among metal workers with special reference to welders. Scand J Work Environ Health.

[b4-ehp0115-a0185b] Navidi W, Lurman F (1995). Measurement error in air pollution exposure assessment. J Exp Anal Environ Epidemiol.

[b5-ehp0115-a0185b] Sokol RZ, Kraft P, Fowler IM, Mamet R, Kim E, Berhane KT (2006). Exposure to environmental ozone alters semen quality. Environ Health Perspect.

